# Endothelial Dysfunction: A Contributor to Adverse Cardiovascular Remodeling and Heart Failure Development in Type 2 Diabetes beyond Accelerated Atherogenesis

**DOI:** 10.3390/jcm9072090

**Published:** 2020-07-03

**Authors:** Aleksandra Gamrat, Michał A. Surdacki, Bernadeta Chyrchel, Andrzej Surdacki

**Affiliations:** 1 Students’ Scientific Group at the Second Department of Cardiology, Jagiellonian University Medical College, 2 Jakubowskiego Street, 30-688 Cracow, Poland; aleksandra.gamrat@gmail.com (A.G.); msurdacki1997@gmail.com (M.A.S.); 2 Second Department of Cardiology, Jagiellonian University Medical College, Jakubowskiego Street, 30-688 Cracow, Poland; chyrchelb@gmail.com

**Keywords:** endothelial dysfunction, type 2 diabetes mellitus, coronary microvascular dysfunction, diastolic dysfunction, heart failure, left ventricular remodeling, large artery stiffening

## Abstract

Endothelial dysfunction, associated with depressed nitric oxide (NO) bioavailability, is a well-recognized contributor to both accelerated atherogenesis and microvascular complications in type 2 diabetes (DM). However, growing evidence points to the comorbidities-driven endothelial dysfunction within coronary microvessels as a key player responsible for left ventricular (LV) diastolic dysfunction, restrictive LV remodeling and heart failure with preserved ejection fraction (HFpEF), the most common form of heart failure in DM. In this review we have described: (1) multiple cellular pathways which may link depressed NO bioavailability to LV diastolic dysfunction and hypertrophy; (2) hemodynamic consequences and prognostic effects of restrictive LV remodeling and combined diastolic and mild systolic LV dysfunction on cardiovascular outcomes in DM and HFpEF, with a focus on the clinical relevance of endothelial dysfunction; (3) novel therapeutic strategies to improve endothelial function in DM. In summary, beyond associations with accelerated atherogenesis and microvascular complications, endothelial dysfunction supplements the multiple interwoven pathways affecting cardiomyocytes, endothelial cells and the extracellular matrix with consequent LV dysfunction in DM patients. The association amongst impaired endothelial function, reduced coronary flow reserve, combined LV diastolic and discrete systolic dysfunction, and low LV stroke volume and preload reserve—all of which are adverse outcome predictors—is a dangerous constellation of inter-related abnormalities, underlying the development of heart failure. Nevertheless, the relevance of endothelial effects of novel drugs in terms of their ability to attenuate cardiovascular remodeling and delay heart failure onset in DM patients remains to be investigated.

## 1. Introduction

Endothelial dysfunction entails depressed bioavailability of nitric oxide (NO), a short-living mediator generated from L-arginine by endothelial NO synthase (eNOS) in response to blood flow-induced shear stress and multiple chemical stimuli [1]. NO diffuses into adjacent vascular myocytes and activates soluble guanylate cyclase with consequent vasorelaxation. Thus, impaired endothelium-mediated arterial vasodilation is a hallmark of endothelial dysfunction [1].

However, in addition to on-going modulation of systemic arterial resistance, NO exerts antioxidant, anti-inflammatory, anti-platelet and anti-thrombotic effects, as well as inhibits migration and proliferation of vascular myocytes, being a key endogenous anti-atherogenic molecule [1]. Endothelial dysfunction is considered an early event in atherogenesis, accompanying atherosclerotic risk factors, including type 2 diabetes (DM) [1,2,3,4,5].

Several mechanisms of reduced NO bioavailability in hyperglycemia and DM have been proposed, evolving from eNOS downregulation by advanced glycation end-products (AGE) [6,7,8,9] into accelerated NO inactivation by superoxide anion, with consequent formation of the more powerful oxidant peroxynitrite, also triggering nitrosative stress and premature endothelial senescence [10,11,12,13]. Elevated superoxide formation in DM results from the protein kinase C-dependent overexpression of membrane-bound NAD(P)H oxidases, mitochondrial superoxide release and so-called eNOS uncoupling, i.e., dissociation of oxygen reduction from L-arginine oxidation by eNOS, which is thereby converted from a NO source into a superoxide generator [13,14,15,16].

Among potential mechanisms of eNOS uncoupling in DM, the oxidation of the eNOS cofactor tetrahydrobiopterin by reactive oxygen species (ROS) appears predominant [17,18], with a possible contribution of the endogenous eNOS antagonist asymmetric dimethylarginine (ADMA) [19,20]. Furthermore, ADMA, stimulating superoxide release from endothelial cells, contributes to an imbalance between NO and ROS [21]. Excessive ADMA accumulation in DM may be mediated by enhanced ADMA synthesis associated with increased protein turnover in insulin-resistant states [22] and the ability of high glucose concentrations to inhibit dimethylarginine dimethylaminohydrolase [23], an enzyme degrading the majority of ADMA [24].

Additionally, DM is associated with increased activity and expression of endothelial arginase I, which decreases the availability of L-arginine to eNOS [25]. Furthermore, the latter mechanism might involve a cross-talk between the endothelium and red blood cells (RBC), where DM—via arginase I up-regulation and eNOS uncoupling—impairs an export of NO bioactivity with consequent endothelial dysfunction [26,27,28]. Notably, enhanced endothelial adhesiveness of RBC presumably promotes these intercellular interactions in DM [28].

Thus, a variety of mechanisms contribute to reduced NO bioavailability in DM. Consequently, long-lasting endothelial dysfunction in epicardial coronary arteries of diabetic subjects precedes the development of atherosclerotic plaques which underlie the vast majority of acute coronary syndromes [29,30].

Regardless of the relevance of ROS overproduction and endothelial dysfunction for both accelerated atherogenesis and microvascular diabetic complications [11,12,31], growing evidence points to impaired endothelial function of the coronary microvessels as a pivotal contributor to diastolic dysfunction and subsequent heart failure with preserved ejection fraction (HFpEF) [32,33], the most common form of HF in DM [34,35,36].

Although myocardial infarction appears to be a likely cause of systolic left ventricular (LV) dysfunction in DM patients with coronary artery disease (CAD) [37], overt CAD develops on a long-term basis in a relatively small proportion of patients with DM [38], whose increasing number has reached epidemic levels worldwide following the closely related obesity [34,39,40]. In particular, CAD prevalence among DM patients was estimated at 21% and a history of myocardial infarction at 10% in a recent extensive review of scientific evidence published between 2007 and 2017 [38]. In contrast, abnormal myocardial function (either asymptomatic or manifesting as symptomatic HF) is more frequent in DM subjects, including also those free of clinically significant CAD. According to a recent position paper of the Heart Failure Association of the European Society of Cardiology (ESC) [35], the prevalence of HF was approximately 20% in clinical trials of glucose-lowering drugs in DM, varying between 4% and 28%. It is noteworthy that DM patients without HF at baseline are 2‒4 times more likely to develop HF over time compared to their non-diabetic counterparts [41,42,43]. In addition, in DM subjects with previously unknown HF, the percentage of newly-recognized with HF was almost five-fold higher for HF with preserved ejection fraction (HFpEF) (about 23%) versus HF with reduced EF (HFrEF) (about 5%) [44]. Moreover, in a large population-based study encompassing 34,198 DM patients participating in the CALIBER program (CArdiovascular disease research using LInked Bespoke studies and Electronic health Records) who were initially free from overt cardiovascular (CV) disease, including CAD, HF was a more common initial presentation of CV disease (866 cases) than non-fatal myocardial infarction (706 cases) over a median follow-up of 5.5 years [45].

Finally, the prevalence of DM in major HF trials was similar in HFrEF and HFpEF, averaging 30–40%, despite the fact that a history of myocardial infarction is usually more common in HFrEF compared to HFpEF [46,47]. Therefore, it is not implausible to assume that the most prevalent causes of HF in DM appear to be mechanisms other than myocardial infarction. These mechanisms are likely to account for an increased risk of HF in DM patients in population-based cohorts [35,41,42,43,48]. Of note, the detrimental prognostic effect of DM in HF subjects was observed irrespective of HF type or CAD prevalence [35]. With regard to HF patients, DM was associated with a higher relative risk of HF hospitalizations or CV death in patients with HFpEF than in patients with HFrEF participating in the CHARM (Candesartan in Heart Failure Assessment of MoRtality and Morbidity) trials [49].

This review is focused on the clinical importance of endothelial dysfunction in DM patients for restrictive left ventricular (LV) remodeling and diastolic dysfunction, predisposing to subsequent development of HFpEF.

## 2. Myocardial Remodeling and Dysfunction in DM—A Two-Faced Disorder

Diabetes mellitus-related myocardial dysfunction and consequent HF can traditionally be considered as a two-faced disorder, corresponding to two different phenotypes of LV remodeling, dilated or restrictive [36]. Almost 50 years ago, it was suggested that DM may be an independent factor causing myocardial dysfunction. Following an earlier observation by Lundbaek [50], the concept of diabetic cardiomyopathy was also proposed by Rubler et al. [51] on the basis of post-mortem findings in four patients with DM, glomerulosclerosis and a history of advanced HF symptoms. Those patients presented eccentric LV hypertrophy, interstitial fibrosis and microvascular wall thickening due to the deposition of acid mucopolysaccharide material in the subendothelial space [51]. As there were no other detectable causes of LV dysfunction beyond DM, that report [51] is consistent with the current definition of cardiomyopathy as “A myocardial disorder in which the heart muscle is structurally and functionally abnormal, in the absence of coronary artery disease, hypertension, valvular disease and congenital heart disease sufficient to cause the observed myocardial abnormality”, proposed by the ESC experts [52].

Nevertheless, although that original report [51] corresponded to dilated cardiomyopathy associated with systolic HF, i.e., HFrEF, restrictive LV remodeling and diastolic LV dysfunction are more frequent in DM [34,35,36], leading to HFpEF. Notably, in major trials and registries of HF patients regardless of HF type (i.e., HFpEF and HFrEF), the coexistence of DM was associated with comparable EF, a similar or even lower LV end-diastolic volume but a higher E-to-e’ ratio, a non-invasive echocardiographic estimate of LV filling pressure [53]. This constellation of findings is consistent with a more restrictive pattern of LV remodeling. Moreover, in some analogy to hypertensive patients who do not commonly progress from concentric to eccentric LV hypertrophy and systolic dysfunction without an interval myocardial infarction [54], a conversion from the restrictive into the dilated phenotype and a more pronounced gradual decline in EF over time was enhanced by older age and CAD, especially interim myocardial infarction [47], but not DM [46] in large cohorts of HF patients.

Therefore, as proposed by Paulus and Dal Canto [53], the similarity of the hemodynamic effects associated with DM in the above-mentioned trials and registries [53] and the lack of the DM-induced shift of HF phenotype from HFpEF into HFrEF [46] suggest common mechanisms by which DM influences LV structure and function irrespective of EF or coexistent CAD.

## 3. Association of Diastolic and Mild LV Systolic Dysfunction in DM

Although HFpEF and HFrEF can be clearly differentiated from each other by the well-established criteria reflecting either a restrictive or dilated phenotype of LV remodeling, diastolic and discrete systolic LV dysfunction frequently coexist in DM, thereby suggesting some overlapping of the two phenotypes.

Faden et al. [55] and Giorda et al. [56] reported a high prevalence of diastolic and subtle systolic dysfunction among asymptomatic subjects with DM, most of whom (96‒97%) had preserved EF, participating in the SHORTWAVE (SHORTening of midWall and longitudinAl left Ventricular fibers in diabetes) and DYDA ((Left ventricular Dysfunction in DiAbetes) multicenter studies. The SHOCKWAVE [55] and DYDA [56] studies recruited DM subjects in sinus rhythm and without clinical evidence of cardiac disease at enrollment, including the lack of inducible myocardial ischemia on exercise electrocardiography or stress echocardiography. Out of 386 SHOCKWAVE study participants, 61 patients (16%) exhibited isolated diastolic dysfunction, 106 subjects (27%) presented with subtle systolic LV dysfunction, while combined diastolic and subtle systolic LV dysfunction was found in 95 patients (25%) [55]. As for the DYDA study, isolated diastolic, discrete systolic and combined LV dysfunction were observed in about 26%, 23% and 11% of a total of 751 subjects, respectively [56]. Diastolic LV dysfunction was defined on the basis of an abnormal mitral inflow pattern in the DYDA study [56], while in the SHOCKWAVE study both transmitral and pulmonary vein flow as well as peak diastolic velocity of the mitral annulus (by tissue Doppler) were taken into account [55].

With regard to systolic LV dysfunction, it was defined as an EF ≤ 50% or LV midwall fractional shortening (mwFS) ≤ 15% in the DYDA study [56], whereas the SHOCKWAVE criterion was either a depressed mwFS below the 10th percentile in healthy controls after correction for individual circumferential end-systolic LV midwall stress (cESS, an index of LV afterload), or an averaged peak systolic velocity of the mitral annulus < 8.5 cm/s by tissue Doppler [55].

In contrast to EF, calculated by tracing of LV endocardial borders by means of the biplane method, mwFS—estimated in the SHOCKWAVE and DYDA studies—represents systolic shortening of the LV short axis at the midwall level. The calculation of mwFS takes into account the migration of midwall myocardial fibers during systole towards the epicardium from the middle line between the endocardium and epicardium, thereby avoiding EF overestimation in patients with concentric LV geometry [57]. Additionally, circumferentially oriented myocardial fibers predominate in the midwall at the LV equator, which enables a proper analysis of the inverse fundamental stress–shortening relationship (i.e., mwFS as a function of cESS). Accordingly, mwFS reflects the shortening of circumferential fibers counteracting cESS, because both cESS and fiber shortening—albeit opposite to each other—are positioned along the same axis, concordant with the most prevalent fiber orientation [57]. Therefore, the adjustment of mwFS for cESS provides an afterload-independent index of circumferential LV systolic function. In contrast, peak systolic velocity of the mitral annulus reflects longitudinal LV systolic function, largely dependent on longitudinal myocardial fibers which predominate in the subendocardial layer. In summary, the described methodology, used by the SHOCKWAVE [55] and DYDA [56] investigators, offers a unique opportunity of complex assessment of both circumferential and longitudinal LV systolic function, adjustment for LV afterload and elimination of a bias resulting from overestimation of LV performance by traditional EF in concentric LV hypertrophy. 

The relevance of the complex estimation of LV function could also be concluded from an earlier work by Andersson et al. [58]. They demonstrated a selective impairment of longitudinal LV systolic and diastolic function (assessed by tissue Doppler mitral annular velocity and strain analysis by speckle-tracking echocardiography) in DM patients free of CAD in comparison with controls matched for age, gender and presence of hypertension despite no identifiable changes in EF and other conventional measures of LV function [58]. In agreement with that observation, Vinereanu et al. [59] reported that the number of major CV risk factors, including DM, was associated with progressive decreases in LV long-axis systolic function, which were balanced by compensatory rises in radial function, thereby maintaining a normal EF. Finally, in asymptomatic DM patients without overt cardiac disease and with controlled blood pressure, Ernande et al. [60], observed subclinical longitudinal systolic dysfunction (assessed by strain analysis) in 28% of DM subjects with normal diastolic function and in 35% of their counterparts with diastolic dysfunction, detected in 47% of the study population.

Hence, keeping in mind the central role of endothelial dysfunction in the novel paradigm for HFpEF [32,33], the aforementioned frequent coexistence of LV diastolic dysfunction with a subtle decrease in LV contractility suggests a probable contribution of coronary microvascular endothelial damage to both these abnormalities.

## 4. Endothelial Dysfunction and Adverse CV Remodeling in DM: Cellular Mechanisms, Hemodynamic Consequences and Clinical Relevance (Table 1)

### 4.1. Cellular Mechanisms of Restrictive LV Remodeling

In contrast to direct cardiomyocyte damage, underlying the dilated phenotype of LV remodeling and HFrEF, multiple comorbidities, including obesity, metabolic syndrome and DM, induce a systemic proinflammatory state with consequent endothelial dysfunction within coronary microvessels. This proinflammatory state appears to be a key player responsible for LV diastolic dysfunction, restrictive LV remodeling and HFpEF [32,33]. Multiple pathways have been described as potential contributors to the association between depressed NO bioavailability in coronary microvessels and impaired diastolic function. These pathways include depressed coronary flow reserve (CFR) [61,62,63], delayed LV active relaxation [64,65] and lower passive LV distensibility [65,66,67,68] due to troponin I [64,65] and titin [66,67,68] protein kinase G-dependent hypophosphorylation. Furthermore, microvascular endothelial dysfunction promotes LV hypertrophy with enlarged cardiomyocytes through the parallel addition of sarcomeres because of the removal of a NO-dependent brake on pro-hypertrophic stimuli [32,69,70]. Additionally, endothelial activation is associated with increased expression of adhesion molecules and local infiltration and accumulation of macrophages which express tumor growth factor-β, thereby stimulating transformation of myocardial fibroblasts into myofibroblasts with consequent reactive interstitial fibrosis [32,53].

### 4.2. Hemodynamic Consequences of Restrictive LV Remodeling in DM

The above described abnormalities enhance restrictive LV remodeling, i.e., a small and stiff ventricle with a consequent fall in stroke volume (SV) in DM [32,34,35,36,53]. Accordingly, in DM patients with the restrictive LV remodeling phenotype, the ability to adjust cardiac output to metabolic demands via recruitment of LV preload reserve (i.e., LV dilatation and activation of the Frank–Starling mechanism) is limited [65]. Therefore, accelerated heart rate (HR) becomes the only way of adjusting cardiac output to increased demand, thereby reducing the duration of diastole, which further aggravates the already impaired LV filling and predisposes to pulmonary congestion and overt HF symptoms.

Importantly, low stroke volume index (SVI) (i.e., below 19th percentile) was associated with an increase of over 80% in the incidence HF among the Strong Heart Study participants [71], including a population-based cohort of 2885 American Indians (47% with DM) with preserved EF and without prevalent HF. Over a median follow-up of 12 years, both low SVI and elevated baseline HR were independent predictors of future HF, regardless of other confounders, including even interim myocardial infarction as a competing risk event.

Therefore, the link between endothelial dysfunction and restrictive LV remodeling with decreased SV might also imply an association between impaired endothelial function and the risk of developing symptomatic HF. Moreover, as even mild coexistent systolic dysfunction further decreases SVI, the low SVI‒HF risk association could even be strengthened by the coincidence of diastolic and discrete systolic LV dysfunction. Concomitant subtle systolic LV dysfunction was observed in 30‒61% of diabetics with LV diastolic dysfunction in the SHOCKWAVE and DYDA studies [55,56], while the respective proportion averaged 35% in the study by Ernande et al. [60] and was as high as 83% in HFpEF subjects (62% with hypertension, 12% with DM) participating in the CARRY-IN-HFpEF (Cardiac Geometry and Function in Heart Failure With Preserved Left Ventricular Ejection Fraction) study [72]. This detrimental association could also contribute to the best prognosis of DM patients with both preserved LV diastolic function and without even subtle LV systolic dysfunction [73].

### 4.3. Clinical Relevance of Endothelial Dysfunction in HFpEF and DM

Notably, as early as 2008, Kistorp et al. [74] described elevated circulating biomarkers of endothelial dysfunction (von Willebrand factor and E-selectin) and their relation to prognosis in HF patients with DM in contrast to nondiabetic HF subjects; however, they studied only patients with EF ≤ 45%. Nevertheless, markers of endothelial dysfunction were later shown to associate with the presence of HFpEF [75,76,77], with the degree of abnormalities in LV diastolic function [78] and even predicted adverse CV outcome [79] in prospective studies of patients with HFpEF. Of note, CFR, largely determined by the endothelial function of coronary microvessels and depressed in HFpEF [61,62,63], was related to the magnitude of peripheral endothelial dysfunction in the PROMIS-HFpEF study (prevalence and correlates of coronary microvascular dysfunction in heart failure with preserved ejection fraction) patients with HFpEF, out of whom about 25–30% were diabetics [63]. Moreover, in that study, depressed CFR was also independently associated with lower SV and impaired LV systolic function along the longitudinal axis, despite preserved EF [63]. It is noteworthy that decreased CFR predicts adverse CV events in patients with normal coronary arteries or non-obstructive CAD [80].

Accordingly, the association among reduced CFR, impaired endothelial function, LV diastolic and discrete systolic dysfunction and low SVI—all of which are adverse CV outcome predictors [29,30,71,73,79,80] —is a dangerous constellation of inter-related abnormalities, jointly contributing to the development of symptomatic HF and HF-related events. In particular, Galderisi [61] proposed that systolic dysfunction might be also a consequence of both microvascular abnormalities and diastolic dysfunction, sharing multiple underlying mechanisms, such as insulin resistance, hyperglycemia, endothelial dysfunction, sympathetic overdrive and hyperactive renin-angiotensin axis.

### 4.4. Mechanisms of Combined Diastolic and Systolic LV Dysfunction in DM

Among the candidate mechanisms of this association, it can be proposed that they are likely to be shared by the pathways implicated in both LV diastolic and systolic dysfunction, responsible for the development of HFpEF and HFrEF, respectively, as elegantly reviewed by Paulus and Dal Canto [53]. Accordingly, AGE can accumulate in coronary microvessels—contributing to excessive oxidative stress and depressed NO bioavailability, delayed active LV relaxation, passive cardiomyocyte stiffening and hypertrophy, as well as microvascular inflammation and reactive interstitial fibrosis with collagen deposition in-between cardiomyocytes [53]. Furthermore, AGE deposition was reported in a close proximity to cardiomyocytes [81], with deleterious effects on systolic function via NAD(P)H oxidase and nuclear factor-κB activation and subsequent apoptotic cell death [53,82]. Additionally, prolonged hyperglycemia, responsible for AGE formation, is a potent stimulus of protein kinase C, accountable for myocardial replacement fibrosis accompanying HFrEF [53].

Likewise, lipid overload and lipotoxicity—dependent on excessive accumulation of triglycerides, diacylglycerols and ceramides, ROS formation, mitochondrial respiratory dysfunction and impaired calcium handling—can lead to cardiomyocyte cell death and HFrEF [53,83,84,85]. Nevertheless, lipotoxicity can exert deleterious effects also on the endothelial level [36,86], thereby contributing to diastolic dysfunction. Interestingly, diacylglycerols and ceramides impair insulin signaling (via protein kinase C activation and phosphoinositide 3-kinase/protein kinase B (Akt) inhibition) and NO bioavailability, in part by inducing eNOS uncoupling [36,83,84,85,86]. Notably, a human study revealed an association between myocardial triglyceride content and LV diastolic dysfunction in uncomplicated DM [87].

Furthermore, in insulin-resistant DM subjects, increased delivery of free fatty acids further suppresses glucose oxidation—in agreement with the Randle cycle-mediated competition between free fatty acids and glucose oxidation—and potentiates insulin resistance which, in turn, contributes to oxidative stress, reduced NO bioavailability and cardiomyocyte hypertrophy [36,53,83,84,85,86]. Additionally, potentiated utilization of free fatty acids as a preferential myocardial substrate is associated with less formation of adenosine triphosphate (ATP) per oxygen molecule [88]. Moreover, a potentiated reliance of cardiomyocytes on fatty acid oxidation can enhance mitochondrial formation of ROS, with impaired oxidative phosphorylation and increased production of heat instead of ATP [36,53,83,84,85,86]. 

Finally, coronary microvascular rarefaction, i.e., reduced capillary density relative to cardiomyocyte surface area, might facilitate both diastolic dysfunction (via reduced NO availability) and systolic impairment through exacerbated subendocardial ischemia [35,36,53].

### 4.5. Large Artery Stiffening—A Contributor to Adverse CV Remodeling in DM

In addition to intrinsic myocardial abnormalities and limited LV preload reserve, the depression of LV performance in DM can be enhanced by excessive afterload. Beyond hypertension, frequently coinciding with obesity and DM, large artery stiffening may also contribute to increased afterload. Due to decreased arterial compliance, the arterial pulse wave travels faster, which allows the reflected wave to return to the aorta earlier, thereby increasing central systolic pressure and lowering diastolic pressure. Among mechanisms accountable for arterial stiffening in DM, augmented collagen synthesis and cross-linking due to AGE accumulation appear of paramount importance [89]. Notably, depressed NO bioavailability within the arterial tree is also likely to contribute to these abnormalities [89]. In particular, not only can AGE depress NO synthesis and bioavailability via its quenching [6,7,8,9], but also NO decreases AGE formation [90], inhibits extracellular matrix production [91] and the proliferation of vascular myocytes [92]. Thus, the excessive pulsatile component of the LV afterload seems to be another detrimental effect of endothelial dysfunction in DM, regardless of its well-recognized effects, i.e., accelerated atherogenesis and higher peripheral vascular resistance.

The similarity of abnormalities at the vascular and cardiac level might also contribute to common associations of cardiac and vascular changes in DM irrespective of concomitant diseases. Indeed, about 20 years ago, Strong Heart Study investigators observed both depressed systemic arterial compliance and subtle LV systolic dysfunction despite preserved EF in 1810 diabetic American Indians compared to their 944 nondiabetic counterparts [93]. Moreover, they also described additive contributions of DM and hypertension to these abnormalities [94]. Additionally, in a retrospective observational study, our group reported both load-independent discrete depression of LV systolic performance (estimated by means of the mwFS‒cESS relationship), elevated LV afterload and lower systemic arterial compliance in patients with aortic stenosis and concomitant DM compared to their nondiabetic counterparts [95] (Table 1).

## 5. Novel Therapeutic Strategies to Improve Endothelial Function in DM

Statins and inhibitors of the renin-angiotensin axis have demonstrated the capability to prevent eNOS uncoupling, improve NO bioavailability and attenuate endothelial dysfunction [96,97,98]. Among antidiabetic drugs, the evidence of endothelial benefits appears strongest for metformin, although negative clinical studies also were published [99,100,101,102,103]. Of note, treatment with metformin for 12 months reduced LV mass (by magnetic resonance imaging) and oxidative stress in a recent small, double-blind, placebo-controlled study of nondiabetic CAD patients with LV hypertrophy, EF ≥ 45% and prediabetes or insulin resistance [104].

Recently, sodium-glucose cotransporter-2 inhibitors, known to potently and rapidly reduce the risk of developing HF and HF-related events in DM [105,106,107], were reportedly shown to correct endothelial dysfunction [108,109], improve large artery compliance [108,110], prevent LV hypertrophy [109] and diastolic dysfunction [111]. With regard to glucagon-like peptide-1 receptor agonists, whose clinical benefit consists in a reduced risk of three-point major adverse CV events, but not HF-related endpoints [37,112], their endothelial effects in human studies remain controversial [103].

Among possible future treatment targets aimed at the correction of endothelial dysfunction in DM, arginase I appears potentially attractive. As mentioned previously, arginase I upregulation in RBC of DM patients may lead to eNOS uncoupling in RBC with consequent ROS formation, which can translate into the dysfunction of adjacent endothelium [25,26,27,28]. Notably, RBC isolated from DM patients impaired endothelium-dependent dilation of isolated vascular segments [27]. Furthermore, in an experimental model of myocardial ischemia-reperfusion injury, the administration of RBC from DM subjects to isolated rat hearts increased infarct size and impaired post-ischemic recovery of cardiac function compared to RBC from healthy controls [26]. Importantly, both of the effects [26,27] could be prevented by arginase blockade.

Finally, clinical reports also suggest potential benefits of arginase inhibition in DM. First, arginase blockade improved endothelial function in DM patients [113,114,115]. Second, the concept of arginase overexpression might also explain the findings from the 4D study (Deutsche Diabetes Dialyse Studie) [116]. In 1244 DM patients on maintenance hemodialysis, März et al. [116] observed the association of low circulating L-homoarginine—degraded by arginases to L-lysine [117]—with increased CV mortality risk and a decreased arginine-to-ornithine ratio, which could indirectly suggest a higher arginase activity [116]. Nevertheless, large prospective clinical studies are warranted to prove benefits of arginase inhibitors in DM subjects.

## 6. Conclusions

Beyond associations with accelerated atherogenesis [37,38], microvascular diabetic complications [12,31] and deterioration of glucose tolerance [118,119,120], endothelial dysfunction supplements the multiple interwoven pathways affecting cardiomyocytes, vascular endothelial cells and the extracellular matrix with consequent impairment of LV diastolic and systolic function in DM. The association among impaired endothelial function, reduced CFR, combined LV diastolic and discrete systolic dysfunction, and low LV stroke volume and preload reserve—all of which are adverse CV outcome predictors—is a dangerous constellation of inter-related abnormalities, underlying the development of HF. Nevertheless, the relevance of endothelial effects of novel drugs in terms of their ability to attenuate CV remodeling and delay HF onset in DM patients remains to be investigated.

## Figures and Tables

**Table 1:** Consequences of endothelial dysfunction in patients with DM.

Cause	Mechanisms	Effects
Low NO bioavailability in coronary microvessels Increased myocardial ROS formation	Titin hypophosphorylation [66,67,68]	Increased cardiomyocyte passive stiffness
	Troponin I hypophosphorylation [64,65]	Delayed/slower active relaxation
	Decreased CFR [61,62,63] and capillary rarefaction [36,53] with consequent subendocardial ischemia	
	Decreased CFR [61,62,63] and capillary rarefaction [36,53] with consequent subendocardial ischemia	Subtle decrease in LV contractility
	Lipotoxicity [83,84,85,86,87,88] Mitochondrial dysfunction [13,36,53]	
	Enhanced tumor growth factor signaling [32,33,53] Renin-angiotensin axis activation [13] Insulin resistance [36,53]	Cardiomyocyte enlargement Interstitial myocardial fibrosis
Restrictive LV remodeling	Cardiomyocyte enlargement [36,53,69,70] Interstitial myocardial fibrosis [34,36,53]	Concentric LV hypertrophy
	Delayed/slower LV relaxation [64,65]	Delayed onset of LV filling
	Increased cardiomyocyte passive stiffness [32,36,53,68] Interstitial myocardial fibrosis [34,36,53]	Decreased LV compliance
	Subtle decrease in LV contractility [34,55,56,58,59,60]	Longitudinal and circumferential LV systolic dysfunction with preserved EF
	Decreased LV compliance [34,53,73] Concentric LV hypertrophy [34,53,93]	Decreased LV preload reserve
Increased collagen deposition and cross-linking in the wall of large arteries	Decreased systemic arterial compliance [89,93] Accelerated pulse wave propagation [89] with consequent earlier return of reflected wave to the aorta, increase in central systolic pressure and lower diastolic pressure	Increased LV afterload Reduced coronary perfusion

CFR: coronary flow reserve; DM: type 2 diabetes mellitus; EF: ejection fraction; LV: left ventricular; NO: nitric oxide; ROS: reactive oxygen species.
